# Congenital Syngnathia With Holoprosencephaly: A Case Report of a Fatal Presentation in a Resource‐Limited Setting

**DOI:** 10.1002/ccr3.71659

**Published:** 2025-12-09

**Authors:** Asteway M. Haile, Biruk T. Mengistie, Tinsae Y. Tamiru, Chernet T. Mengistie, Dawit G. Abebe, Bezawit M. Haile

**Affiliations:** ^1^ School of Medicine, College of Health Sciences Addis Ababa University Addis Ababa Ethiopia; ^2^ Pediatrics Department ALERT Comprehensive Specialized Hospital Addis Ababa Ethiopia; ^3^ Department of Pediatrics and Child Health, College of Health Sciences Addis Ababa University Addis Ababa Ethiopia

**Keywords:** case report, congenital syngnathia, holoprosencephaly, neonatal mortality, resource limited settings

## Abstract

Congenital syngnathia, the rare fusion of the maxilla and mandible, poses significant feeding and respiratory challenges. Its management is complicated by rarity and potential syndromic associations. We present a 1‐week‐old female neonate who presented with left jaw deviation, respiratory distress (SpO_2_ 69%, tachypnea), and fever. Examination revealed near‐complete inability to open the mouth except for a small right paramedical gap. A CT scan confirmed left maxilla‐mandibular bony fusion (Laster type 2b, Dawson type 2b), mandibular hypoplasia, semilobar holoprosencephaly, corpus callosal dysgenesis, a dorsal cyst, and microcephaly. Concurrent neonatal sepsis was diagnosed. Management included antibiotics, NG tube feeding, and oxygen support. Surgical intervention was deferred due to critical instability. Despite supportive care, the infant's condition deteriorated, resulting in fatal respiratory failure. This case underscores the lethal potential of congenital syngnathia when compounded by severe CNS malformations. The CNS anomalies induced hypoventilation and aspiration, making surgery nonviable and leading to sepsis and respiratory failure. The absence of antenatal care prevented early detection and tertiary delivery planning. Prompt neuroimaging is crucial, and prognosis is heavily dictated by CNS comorbidities, necessitating consideration of palliative prioritization in resource‐limited settings.

## Key Clinical Message

Congenital syngnathia is a rare but life‐threatening condition, especially when compounded by severe central nervous system malformations. Early neuroimaging, antenatal detection, and multidisciplinary planning are critical.

## 1 Background

Congenital syngnathia is a rare disorder of the craniofacial region characterized by complete or partial fusion of the upper and lower jaws. It can involve a single mucosal band (synechiae) or maxillary and mandibular bones (complete synostosis). Davis reported the first case of synechia in 1935 [1] whereas the first case of syngnathia was reported by Burket [2]. Syngnathia can occur either along the midline or laterally and may be unilateral or bilateral. Most documented cases involve a unilateral fibrous fusion [3, 4]. It mainly affects the opening of the mouth, causing difficulty in feeding and respiration. Syngnathia can occur as an isolated condition or be associated with various syndromes. Acquired syngnathia has been documented as a complication of myositis ossificans or other conditions that restrict mouth opening [5]. Several classification systems for congenital syngnathia have been proposed over the years, focusing on treatment outcomes, location, tissue fusion, and syndromic associations. There has been no standard treatment for this condition due to its rarity, and few patients live without surgical intervention.

## 2 Clinical History/Examination

A 1‐week‐old female neonate was referred to our hospital with complaints of difficulty opening the mouth and associated respiratory distress. She was delivered vaginally at home to a 28‐year‐old mother, gravida III para III, who had not attended any antenatal care follow‐ups. There was no history of trauma, consanguinity, or similar conditions in the family. On presentation, the neonate was febrile with a recorded temperature of 38.3°C and had severe hypoxemia with an oxygen saturation of 69% on room air, indicating significant respiratory compromise. She was tachypneic, with a respiratory rate of 70 breaths per minute, and exhibited subcostal retractions. Examination also revealed oral secretions pooling in the mouth, a noticeable deviation of the face to the left with obliteration of the right nasolabial fold, and low‐set ears, though no other external anomalies were apparent. Importantly, the neonate was unable to open her mouth except for a small opening at the right paramedial region.

## 3 Differential Diagnosis, Investigations and Treatment

**FIGURE 1:**
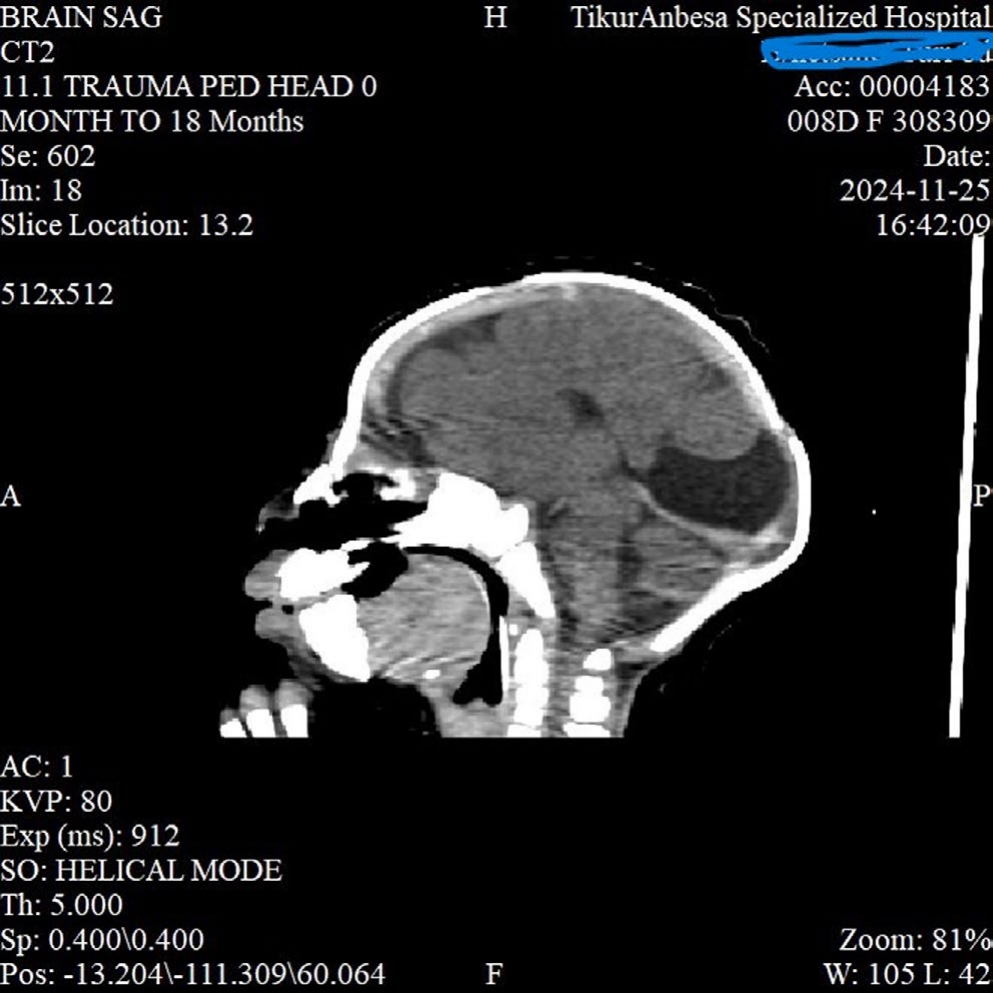
Sagittal non‐contrast CT of the head showing severe craniofacial dysmorphism, including abnormal anterior skull base configuration and markedly malformed midface structures. The oral cavity and mandible appear abnormally positioned, and there is distortion of the nasal and pharyngeal spaces. No normal segmentation of facial structures is seen, consistent with complex congenital craniofacial malformation. This figure illustrates left maxilo‐mandibulary bony fusion, left mandibular hypoplasia and central incisior, semilobar holoprosencephaly with corpus callosal dysgenesis, dorsal interhemispheric cyst and microcephaly.

**FIGURE 2:**
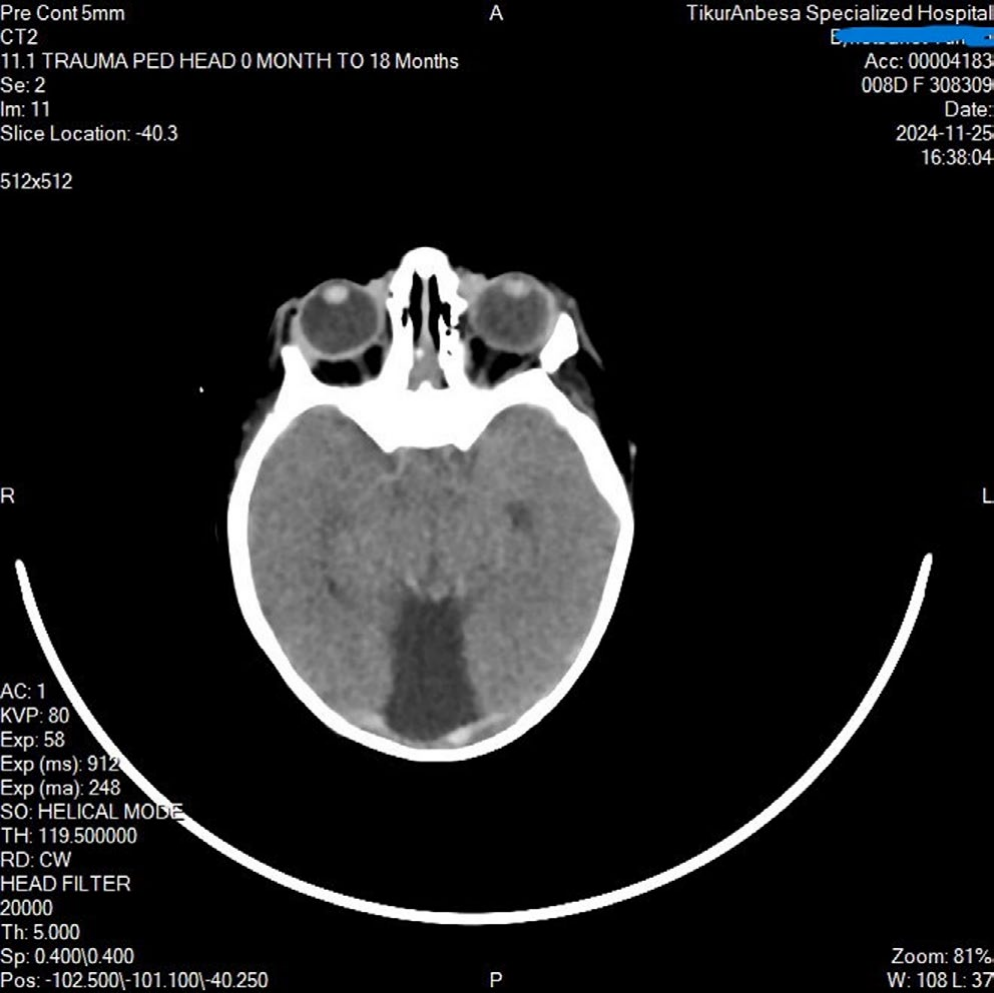
Axial non‐contrast CT of the brain demonstrating markedly abnormal cerebral architecture. There is fusion of the frontal lobes with absence of normal midline separation and a single large forebrain cavity, suggestive of holoprosencephaly‐type malformation. Orbits are present but show abnormal spacing relative to midline structures. This figure illustrates left maxilo‐mandibulary bony fusion, left mandibular hypoplasia and central incisior, semilobar holoprosencephaly with corpus callosal dysgenesis, dorsal interhemispheric cyst and microcephaly.

**FIGURE 3:**
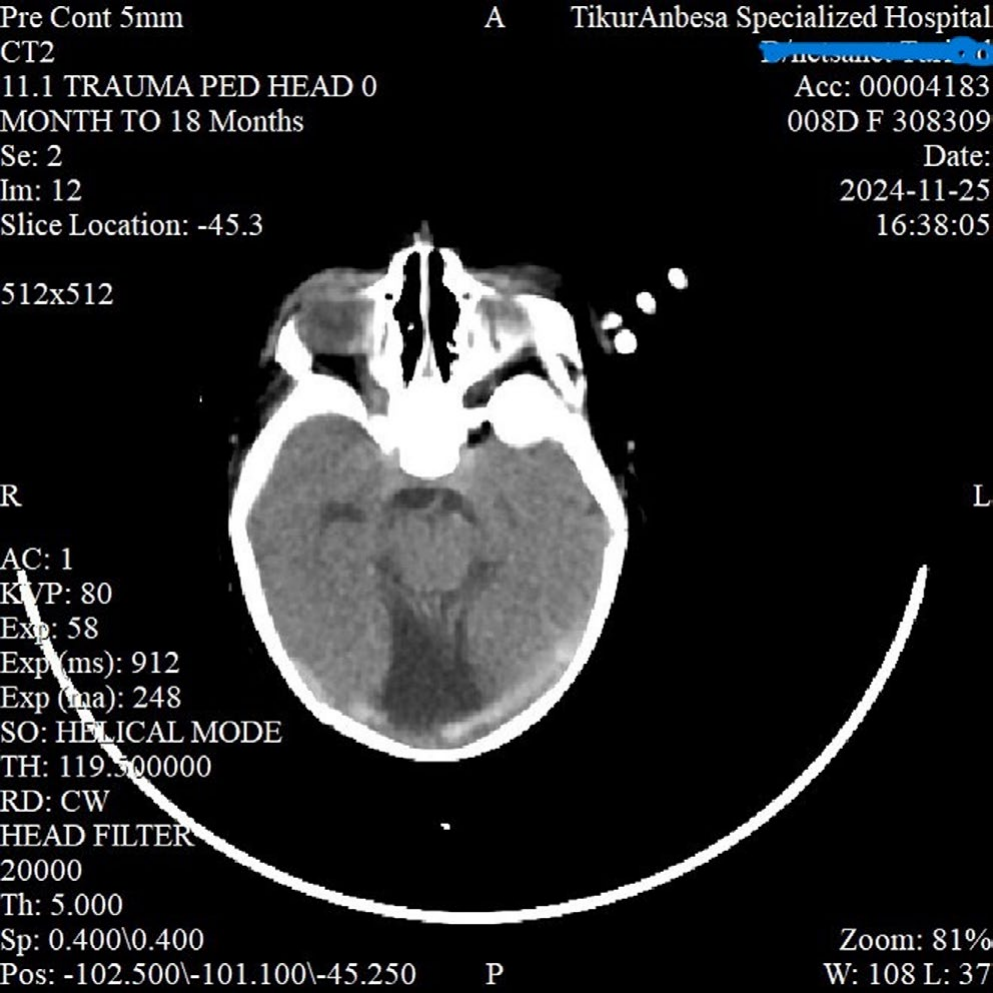
Axial non‐contrast CT showing poorly formed midline structures and a single ventricle–like cavity. The nasal cavity and anterior skull base are malformed, with irregular bony margins. This figure illustrates left maxilo‐mandibulary bony fusion, left mandibular hypoplasia and central incisior, semilobar holoprosencephaly with corpus callosal dysgenesis, dorsal interhemispheric cyst and microcephaly.

Given the inability to open the mouth, jaw deviation, and associated respiratory distress, congenital syngnathia (maxillomandibular fusion) was considered the most likely diagnosis. Other craniofacial syndromes associated with mandibular anomalies, such as Treacher Collins syndrome or Pierre Robin sequence, were also entertained but considered less likely in view of the evident bony fusion. The presence of fever and systemic illness raised suspicion for late‐onset neonatal sepsis, which was later confirmed through positive blood cultures. To further delineate the extent of craniofacial involvement, a CT scan of the head (Figure 1) and face was performed, which revealed left maxillo‐mandibular bony fusion, left mandibular hypoplasia (Figure 2), semilobar holoprosencephaly with corpus callosal dysgenesis (Figure 3), a dorsal interhemispheric cyst, and microcephaly. According to Dawson's classification, the patient was categorized as type 2b, and by Laster's classification, also type 2b.

Management was initiated promptly with intravenous antibiotic therapy for sepsis, alongside supportive measures such as nasogastric tube feeding for nutrition and intranasal oxygen supplementation to relieve respiratory distress. Given the complexity of the case, a multidisciplinary approach was adopted to address both the craniofacial fusion and intracranial anomalies. Although surgical separation of the fused structures was considered, the intervention was deferred due to the neonate's critical condition and poor overall prognosis. Supportive and conservative care remained the mainstay of management.

## 4 Outcome and Follow‐Up

Despite appropriate medical care and supportive interventions, the infant's condition progressively worsened. The combination of severe craniofacial malformation, multiple intracranial anomalies, and sepsis contributed to a grave prognosis. Ultimately, the neonate succumbed to respiratory failure, and surgical correction was not attempted. The case highlights the challenges in diagnosing and managing rare congenital anomalies, the importance of multidisciplinary involvement, and the need for early antenatal detection where possible.

## 5 Discussion

Syngnathia is a rare disorder with variable clinical presentation ranging from partial fusion to complete bony ankylosis of the maxilla to the mandible. Synechiae management is well documented in the literature and typically involves straightforward treatment. However, syngnathia is a rarer deformity than fusion by soft tissue. Most cases of congenital syngnathia are associated with congenital defects such as cleft lip, cleft palate, microglossia, micrognathia, and TMJ disease, with cleft lip being the most common [6]. Other coexisting abnormalities include mandibular clefts, oblique facial clefts, tongue anomalies, glossopalatine ankylosis, mandibular hypoplasia, coloboma, hypophyseal duplication, and frontonasal malformations. There was also a reported case of syngnathia associated with bilateral hemimelia and apodia, highlighting the coexistence of congenital limb and craniofacial anomalies [7] The exact cause of syngnathia is still unclear, and several theories have been suggested, including a diminished fetal swallowing reflex (Humphrey), failure of tongue protrusion, and uncontrolled proliferation of ectoderm [8, 9]. The persistence of the buccopharyngeal membranehas been the most commonly cited cause. Other suggested causes includeamniotic constriction bands in the region of the developing first branchial arch, environmental insults, and the use of drugs such as large doses of meclizine and vitamin A during pregnancy [10, 11]. Laster argued that the deformity is linked to the timing and stage of external damage, noting that external damage occurring after the 12th week of pregnancy does not lead to cleft palate, micrognathism, fusion of the zygomatic bone with the upper jawbone, or fusion of the cortical bones [11].

Even though there is no syndromic presentation, Syngnathia can be found associated with several syndromes like oromandibularlimb hypogenesis syndrome, popliteal pterygium syndrome, aglossia‐adactylia syndrome, Van der Woude syndrome, cleft palate lateral alveolar synechiae syndrome, Nager syndrome, Horner syndrome, and Dobrow syndrome, craniofacial microsomia [12, 13].

In 1997, Dawson proposed the first classification of bony congenital syngnathia in terms of treatment and functional outcome [14]. The classification comprises:

- Type 1 (Simple Syngnathia): bony fusion between mandible and maxilla or zygoma in the absence of other congenital anomalies in the head and neck
- Type 2a (Complex Syngnathia): bony fusion between the mandible and the maxilla or zygoma with aglossia;
- Type 2b (Complex Syngnathia): bony fusion between the mandible and the maxilla or zygoma with agenesis or hypoplasia of the proximal mandible.

Later, in 2001, Laster et al. proposed the following classification for bony syngnathia based on location [11].

- Type 1a (Simple Anterior Syngnathia): bony fusion of the alveolar ridges without other congenital deformities in the head and neck
- Type 1b (Complex Anterior Syngnathia): bony fusion of the alveolar ridges associated with other congenital deformities in the head and neck
- Type 2a (Simple Zygomatico‐Mandibular Syngnathia): bony fusion of the mandible to the zygomatic complex causing mandibular micrognathia;
- Type 2b (Complex Zygomatico‐Mandibular Syngnathia): bony fusion of the mandible to the zygomatic complex and associated clefts or TMJ ankylosis.

Tauro et al. proposed a classification that did not address the nature of the fused tissue, but the location and extension, along with syndromic association in 2012 [15].

In 2020, Olusanya et al. introduced a new classification‐based management protocol comprising [16].

This tragic case demonstrates the lethal interaction between congenital syngnathia and severe central nervous system malformations, an underreported relationship with critical implication for management. The infant's mandibulomaxillary fusion compounded by the CNS malformations led to irreversible respiratory failure. In contrast to isolated congenital syngnathia cases where early surgical intervention may restore function, the profound CNS anomalies here caused hypoventilation making the surgery nonviable. Aspiration resulting from impaired secretion clearance likely caused initiated the fatal septic cascade, accelerated by neurologic compromise. Crucially, the absence of antenatal care prevented prenatal detection of the condition. These missed opportunities could have enabled delivery planning at a tertiary center equipped for immediate airway stabilization. This case establishes the severe CNS comorbidities dictate survival in congenital syngnathia necessitating prompt neuroimaging and palliative prioritization in resource constrained settings.

A prenatal diagnosis can be made via fetal ultrasonography and MRI findings such as micrognathia, the presence of a closed mouth, or restricted fetal mouth movement [4, 17].

Radiographic imaging is important for determining the extent of bony involvement and other involved structures. A 3D reconstructed CT provides a more detailed depiction of the fusion and serves as a guide for surgical planning [14]. A multidisciplinary team and early surgical management of syngnathia are mandatory to avoid respiratory and feeding difficulties and prevent ankylosis of the TMJ. An important step consists of the management of the upper airways, which could be compromised during surgical procedures under general anesthesia. A difficult intubation, because of non‐existent or very limited mouth opening, could be assisted by using a fiberoptic bronchoscope [18]; an elective tracheostomy can be performed and kept in place until the jaw fusion is resolved [19]. It is recommended to use a gastrostomy or insert a nasogastric tube to provide nutrition, particularly in newborns [20].

There is no established management protocol for congenital syngnathia. The condition's rarity and diverse presentations make its management challenging and dependent on the type and severity of the case. Evaluation of the affected infant typically takes into account their current overall health. If survival is likely without urgent surgical intervention, surgery should be postponed to avoid unnecessary risks.

The primary objective of the surgery is to release ankylosis, restore normal mandible function, and prevent recurrence [21]. Surgical exposure depends on the site of fusion [3] and the surgical techniques used are as follows:

1. Soft tissue syngnathia release [22]
2. Bony Syngnathia Release [23]
3. Distraction Osteogenesis [24]
4. Temporomandibular Joint (TMJ) Reconstruction [25].

The prognosis of congenital syngnathia depends on the severity of the condition and the timing of surgical intervention. Mild cases with only soft tissue involvement tend to have excellent outcomes, while severe cases with extensive bony fusion may require multiple surgical interventions and long‐term rehabilitation [26]. The risk of re‐fusion remains a major concern, particularly in cases where interpositional grafts were not placed. However, with early intervention, comprehensive rehabilitation, and adherence to postoperative therapy, most patients achieve functional jaw mobility and improved quality of life.

## 6 Conclusion

Maxillomandibular syngnathia is an exceedingly rare condition of uncertain etiology, presenting with varying levels of severity, ranging from mucosal band fusion (synechia) to complete bony fusion (synostosis) of the jaws. While multiple classification systems have been introduced to assist in diagnosis and surgical planning, there is no universally accepted standard for treatment. However, it is broadly recognized that effective management necessitates a multidisciplinary approach, with early surgical intervention being crucial to secure the patient's airway, ensure proper nutrition, and achieve sufficient mouth opening while preventing recurrence.

## Author Contributions

**Asteway M. Haile:** conceptualization, data curation, formal analysis, project administration, resources, supervision, writing – original draft, writing – review and editing. **Biruk T. Mengistie:** conceptualization, data curation, methodology, project administration, resources. **Tinsae Y. Tamiru:** conceptualization, data curation, supervision, writing – original draft. **Chernet T. Mengistie:** conceptualization, data curation, supervision, writing – review and editing. **Dawit G. Abebe:** conceptualization, data curation, resources, writing – original draft. **Bezawit M. Haile:** conceptualization, data curation, methodology, writing – original draft.

## Funding

The authors have nothing to report.

## Ethics Statement

IRB review and approval was waived for this case report.

## Consent

Written informed consent was obtained from the patient for publication of the case details and accompanying images.

## Conflicts of Interest

The authors declare no conflicts of interest.

## Data Availability

The data underlying the results presented in this work is available within the manuscript.
